# Promoting Factors to Stay at Work Among Employees With Common Mental Health Problems: A Multiple-Stakeholder Concept Mapping Study

**DOI:** 10.3389/fpsyg.2022.815604

**Published:** 2022-05-10

**Authors:** Suzanne G. M. van Hees, Bouwine E. Carlier, Roland W. B. Blonk, Shirley Oomens

**Affiliations:** ^1^Occupation and Health Research Group, HAN University of Applied Sciences, Nijmegen, Netherlands; ^2^Tilburg School of Social and Behavioral Sciences, Tilburg University, Tilburg, Netherlands; ^3^TNO, Leiden, Netherlands; ^4^Optentia, North West University, Vanderbijlpark, South Africa; ^5^Department of Primary and Community Care, Nijmegen School of Occupational Health, Radboudumc, Nijmegen, Netherlands

**Keywords:** mental health, workplace interventions, occupational health services, stay at work, stakeholder participation, concept mapping, leadership

## Abstract

Most individuals affected by common mental health problems are employed and actually working. To promote stay at work by workplace interventions, it is crucial to understand the factors perceived by various workplace stakeholders, and its relative importance. This concept mapping study therefore explores perspectives of employees with common mental health problems (*n* = 18), supervisors (*n* = 17), and occupational health professionals (*n* = 14). Per stakeholder group, participants were interviewed to generate statements. Next, each participant sorted these statements on relatedness and importance. For each group, a concept map was created, using cluster analysis. Finally, focus group discussions were held to refine the maps. The three concept maps resulted in several clustered ideas that stakeholders had in common, grouped by thematic analysis into the following meta-clusters: (A) Employee’s experience of autonomy in work (employee’s responsibility, freedom to exert control, meaningful work), (B) Supervisor support (being proactive, connected, and involved), (C) Ways to match employee’s capacities to work (job accommodations), (D) Safe social climate in workplace (transparent organizational culture, collective responsibility in teams, collegial support), and (E) professional and organizational support, including collaboration with occupational health professionals. Promoting stay at work is a dynamic process that requires joined efforts by workplace stakeholders, in which more attention is needed to the interpersonal dynamics between employer and employee. Above all, a safe and trustful work environment, in which employee’s autonomy, capacities, and needs are addressed by the supervisor, forms a fundamental base to stay at work.

## Introduction

Under certain conditions, staying at work for individuals with common mental health problems (CMHPs) not only contributes to their wellbeing and mental health, but also has positive consequences for employers and society, such as reduction of absenteeism (OECD, 2012; de Vries et al., 2018). As most individuals affected by CMHP are employed and actually working, this phase of staying at work needs more attention (OECD, 2012; Danielsson et al., 2017). Interestingly, nearly 40% of a representative panel of Dutch employers does not know how to help employees with CMHP in the workplace (Janssens et al., 2021). One explanation may be the course of CMHP, in which symptoms appear through implicit or ambiguous signals, usually developing stealthily and slowly. Another explanation could be stigma and lack of openness on the work environment about mental health (Moll, 2014; Brouwers et al., 2020). For both the employer and the employee, those reasons may lead to inability to acknowledge the problem, and therefore, employees continue to use (short-term) compensatory strategies and seek for help too late (van Hees et al., 2021b). Employers remain confused about best practices for workplace mental health interventions, especially on each stakeholders’ role and responsibility (Wagner et al., 2016). Therefore, we need to provide workplace stakeholders with clear directions on ways to enable employees with CMHP to continue working.

The perspectives on what employees with CMHP need to stay at work (SAW) may differ between employee, employer, and occupational health professional (OHP), as shown in earlier research on return to work (Hees et al., 2012; Leyshon and Shaw, 2012; de Vries et al., 2014). This could be because each stakeholder may have different interests and goals. Employers want to prevent long-absenteeism, so they prefer to know what problems the employee faces. Opposing, the employee might prefer to continue working without reporting problems, due to the fear to lose income or career perspective. Moreover, among workplace stakeholders, there may be a hierarchy in power relationships. Therefore, it is important to gain insight into what each stakeholder group in the work context regards as important to SAW for employees with CMHP, including the similarities, differences, and relative importance.

The experience of illness in the workplace has been reported in previous studies regarding employees with physical complaints (de Vries et al., 2012; Bosma et al., 2020; van der Mei et al., 2021), however little among employees with CMHP who continue working (Danielsson et al., 2017; van Hees et al., 2021b). Previous studies on work participation among employees with CMHP show that work functioning and work performance are affected by individual factors (e.g., symptom severity, comorbidity), and work-related factors (e.g., high job demands, lower job control) (Lagerveld et al., 2010; Thisted et al., 2018; van Hees et al., 2021b). Interventions are often focused on individual (psychological or medical) treatment (van Hees et al., 2021b), in which work-focused treatment seems more effective than general treatment (Karlson et al., 2014; Brenninkmeijer et al., 2019). Subsequently, there has been a shift over the last years in the literature from the individual (medical or psychological) treatment of CMHP, toward the integration of interventions in the workplace (Lagerveld et al., 2010; Joyce et al., 2016; Nexo et al., 2018). Despite a growing body of evidence, practice shows that it is challenging to intervene effectively in the workplace where practical guidelines or strategies are scarce (Rycroft-Malone et al., 2004; Nexo et al., 2018; Nielsen et al., 2018; Jetha et al., 2021). Interestingly, according to employees with CMHP, strategies to keep working concern their coping with situations especially in the direct work environment, by attempting to retain a sense of autonomy and by getting the possibility to maneuver and perform in their working life (Danielsson et al., 2017). What in the workplace really enables employees to SAW lies in the complexity of how individual factors and work-related factors interact, that is, underexposed in the current research agenda. Understandably, factors on, for example, communication between employee and employer are often less tangible factors and therefore harder to capture (van Hees et al., 2021a). To unravel the dynamic and complex nature of phenomena such as SAW, novel mixed methods research designs are needed (Boot and Bosma, 2020).

There is no uniform definition of SAW (de Vries et al., 2012; van Hees et al., 2021a). Stay at work while facing mental health issues may be confused with presenteeism, which could be defined in the two following ways: (1) as “employees, despite complaints and ill health that should prompt rest and absence from work, still turning up at their jobs” or (2) as “a reduced performance at work, besides illness” (Ishimaru et al., 2020). For both, if not handled appropriately, it may lead to absenteeism. We defined SAW as a positive work outcome that is to continue working, indicated as no absenteeism or not being absent for more than 50% or no longer than 6 weeks (Lagerveld et al., 2010). Working while facing mental health issues can be used as a means to decrease the severity of CMHP, resulting in an increased chance to SAW (van Hees et al., 2021b). Common mental disorders refer to depression, anxiety disorder, or stress-related disorder (Andersen et al., 2012; Harvey et al., 2017). However, a large number of employees who suffer from CMHPs are undiagnosed and do not receive treatment (Pomaki et al., 2012). We also considered this group of employees at risk of negative work outcomes, as a consequence of psychological complaints. Therefore, we used a relatively broad definition of employees with diagnosed mood, anxiety, or stress-related problems as well as self-reported psychological complaints.

In order to gain more insight into perceived factors that promote SAW among employees with CMHP, we investigated the perspectives of the key workplace stakeholders in the process of SAW: employees, supervisors, and OHPs.

## Materials and Methods

### Study Design

This study uses a concept mapping approach. Concept mapping is a structured conceptualization method, designed to organize and represent perspectives regarding one theme of interest (Trochim and McLinden, 2017). It combines information from qualitative data collection and group discussions with multivariate statistical analyses, that, as a mixed methods approach, may be more suitable to capture the complexity of SAW (Andersen et al., 2012; Boot and Bosma, 2020). Concept mapping facilitates a group of individuals to describe their views and represent these visually into a map of clustered factors (Kane and Trochim, 2007). As a diverse group of participants is recommended (Trochim and McLinden, 2017; van Bon-Martens et al., 2017), we explored promoting factors from various perspectives by including three key workplace stakeholder groups. As SAW among employees with CMHP is also a precarious process, in which stakeholders may have different and possibly conflicting interests, we decided to let participants follow the concept mapping steps among peers. This enabled each stakeholder group to collectively represent their perspective regarding the same theme of interest, assuring the generation of valid and reliable results of similarities and differences by facilitating an egalitarian participation of each group (Rosas and Kane, 2012).

### Participants

#### Selection of Participants

Participants were purposefully sampled from the three key stakeholder groups involved in the SAW process, leading to a convenience sample (Trochim and McLinden, 2017). We recruited employees through various strategies, by placing an announcement on the website or in the waiting room of various mental health services (*n* = 4) and activism organizations representing people with CMHP^1^ (*n* = 7), by posting an announcement on social media (*n* = 3) and by distributing flyers at regional employers (*n* = 4). We recruited supervisors by (1) contacting the regional largest employers, (2) placing an announcement on the website of the association representing middle and small-sized employers and the national employer’s association^2^, and (3) posting a short recruitment video on social media. Lastly, we recruited OHPs by posting an announcement on social media and on the website of the national association of labor experts^3^ and large occupational health service practices.

Employees were included based on self-reported CMHP and their current work status (working at least 50% of their contract and thus currently working). Supervisors were included in this study if they are currently working as a direct supervisor and dealt with at least one employee with self-reported CMHP who stayed at work instead of being sick listed. Supervisors and employees were not working at the same organization. As this study is part of a larger (PhD)project to advance labor expertise in prevention of long-term absenteeism, we involved OHPs who are trained as so-called “labor experts” in the Dutch social security system. Labor experts play a key role in supporting the reintegration process of persons with a work disability and remaining work ability. These OHPs are expert in the assessment and interventions needed in return to work process, matching the employee’s capacities with work and work environment. In current practice in Netherlands, the role of labor experts focuses mainly on work reintegration. Recently, the center of labor expertise^2^ aims to explore the rather new role of these labor experts in prevention. Therefore, we included two groups among OHPs: one group with labor experts only and one group with a mix of OHPs, namely, occupational health physicians, occupational health social workers, labor experts, and organizational health advisors. Because this last group proved to have more practical experience with employees with CMHP while staying at work instead of being sick listed, we presented results of the mixed group of OHPs in this paper.

#### Participant Characteristics

A total of 49 participants took part of this study, of whom 18 employees with CMHP, 17 supervisors, and 14 OHPs, referring to Table 1. Most employees with CMHP were highly educated and they were all native Dutch. Most of the supervisors and employees worked in the public sector (healthcare, education, civil services) as well as all OHPs and the type of employees were mostly professionals (e.g., engineer, accountant, system analyst, doctor, nurse, teacher). Employees with CMHP reported to experience mostly stress-related complaints, followed by mood disorders and anxiety.

**TABLE 1:** Participant characteristics.

Characteristic	Employees (*n* = 18)	Supervisors (*n* = 17)	OHPs (*n* = 14)
Age (M)	29–61 (42)	31–62 (48)	46–67 (52.9)
Female (%)	67	56	50
Educational level Medium	2	0	0
Educational level High	16	17	14
ears of work experience (M)	n/a	1–20 (7)	4–35 (15)
type of CMHP-related complaints, self-reported	Stress:15 Mood/depression: 11 Anxiety: 7 More than one: 11	n/a	n/a
Employee type (Hilton et al., 2008)	Executive, administrator or senior manager: 2 Professional (e.g., engineer, accountant, system analyst, doctor, nurse, teacher): 10 Technical support: 1 Sales: 1 Clerical/administrative support: 2 Service occupation: 2		
Sector	Public sector: 14 education (5), healthcare (3), civil services (6) Private sector: 4	Public sector: 14 education (2), healthcare (5), police (4), municipality (3) Private sector: 3	All in public sector

### Data Collection and Analysis

The process of data collection and analysis contained of the following five-steps that were undertaken for each stakeholder group. Data were collected in 2019.

#### Step 1: Focus Question

The first step consisted of the formulation of a focus question to obtain ideas about the topic of interest (Kane and Trochim, 2007). Our focus question was “What employees with CMHP need to enable them to continue working is…” The comprehensiveness of the focus question (step 1), the interviewing of participants to generate statements (step 2), and the feasibility of the online assignment (step 3) were pilot tested among a random group of OHPs and among colleagues of the researchers.

#### Step 2: Generation of Statements

Individual interviews to generate statements (short phrases or sentences reflecting ideas about the topic of interest) were conducted with each participant (Kane and Trochim, 2007). Each interview was conducted either face to face or by telephone and took approximately 30 min. The interviews were executed by one researcher (XX), and the first two interviews for each group were reviewed by a second researcher (XX). Each participant was first given a “warm-up-question” to ensure the focus on the phase of “staying at work” instead of being sick listed, and the target population (employees with CMHP). After this, participants were encouraged to brainstorm and mention as many ideas as possible. The interviewer asked questions related to why and how, in order to concretize the statements on promoting factors to SAW. Each interview was audiotaped and transcribed. Thereafter, one researcher (XX) extracted all statements from each transcript. Statements with similar meanings of the content were merged. The process of extraction and merging of statements was checked by a second researcher (XX) and a third researcher (XX). Saturation occurred in the last interviews of each stakeholder group, that is the main criterion for the number of participants in this step of statement generation (Kane and Trochim, 2007). Most participants were involved in this step (employees: *n* = 17, supervisors: *n* = 16, OHPs: *n* = 13, respectively). Three participants canceled the interview due to time constraints, yet they participated in the other steps.

#### Step 3: Prioritization and Clustering

For each stakeholder group, one researcher (SH) inserted all statements into the statistical program ARIADNE, a web-driven tool specifically designed to support prioritization and clustering in step 3 and statistical analysis in step 4 (NcGv/Talcott, 1995). First, each participant was asked to prioritize each statement by using a 5-point Likert scale of 1 (lowest importance) to 5 (highest importance), distributing all statements equally among the five scales. Second, each participant was asked to cluster the statements with similar content, using at least 2 – but no more than 10 clusters. In total, all but one participant completed this step (employees: *n* = 18, supervisors: *n* = 17, OHPs: *n* = 13, respectively).

#### Step 4: Statistical Analysis

The statistical program Ariadne was used for the data analyses (NcGv/Talcott, 1995). First, the arithmetic mean score assigned to each statement was calculated among participants from each stakeholder group, resulting in a mean importance score of each statement. Second, a multidimensional scaling followed by hierarchical cluster analyses was used on the basis of a matrix of the clustering results (i.e., how often two statements were placed together in the same cluster by participants). This resulted into a two-dimensional visual map for each stakeholder group with a final set of clusters (van Bon-Martens et al., 2017).

#### Step 5: Interpretation of the Concept Maps

Two researchers (XX and XX) independently determined the number of clusters for each concept map and discussed if more or less clusters represented participant’s statements better, by evaluating the relatedness between statements in each cluster. For every stakeholder group, a 2-h focus group session, facilitated by two researchers (XX and XX), was held to interpret their concept map. By critically reviewing the statements covering the clusters, participants named and refined each cluster, in order to represent its content. Through group discussion, some statements were moved to another cluster or clusters were removed, until consensus was reached. Each stakeholder group concluded their reflection with the identification of meta-clusters, and its practical implications. The researchers ultimately selected meta-clusters that the different stakeholder groups had in common through a thematic analysis. Because each stakeholder group created their own map, importance scores across all clusters could not be evaluated. Of note, 40 out of 49 participants who carried out the prioritization and clustering task also participated in the focus group discussions (employees: *n* = 13, supervisors: *n* = 16, OHPs: *n* = 11, respectively).

## Results

First, we presented each stakeholders’ concept map by showing the retrieved clusters and most important statements of promoting factors to SAW. Then, we presented five meta-clusters based on the clusters that stakeholder groups have in common. Finally, we presented the differences between stakeholder perspectives, retrieved through the concept maps and focus group discussions.

### Concept Maps per Stakeholder Group

**FIGURE 1:**
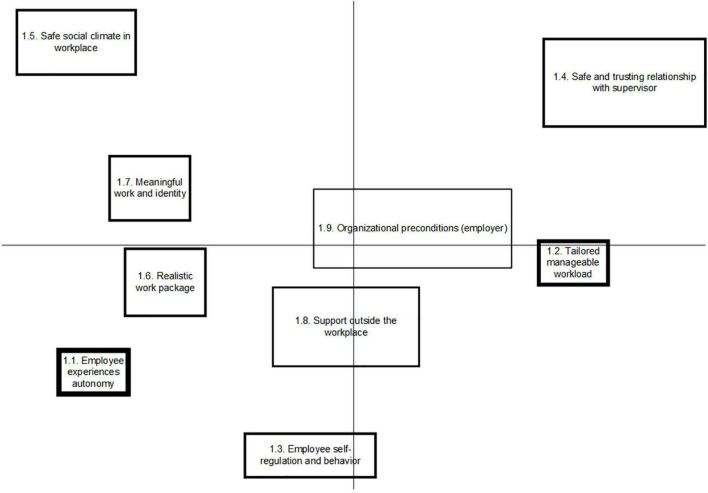
Concept map on factors promoting Stay at work, representing the perspective of employees with CMHP.

**FIGURE 2:**
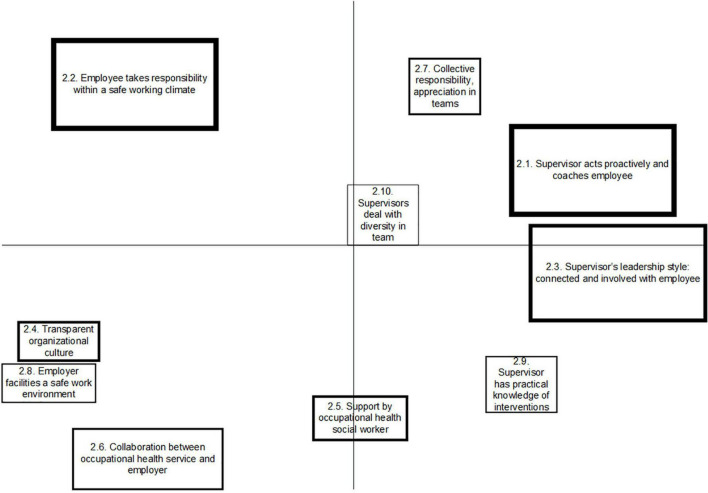
Concept map on factors promoting Stay at work, representing the perspective of supervisors.

**FIGURE 3:**
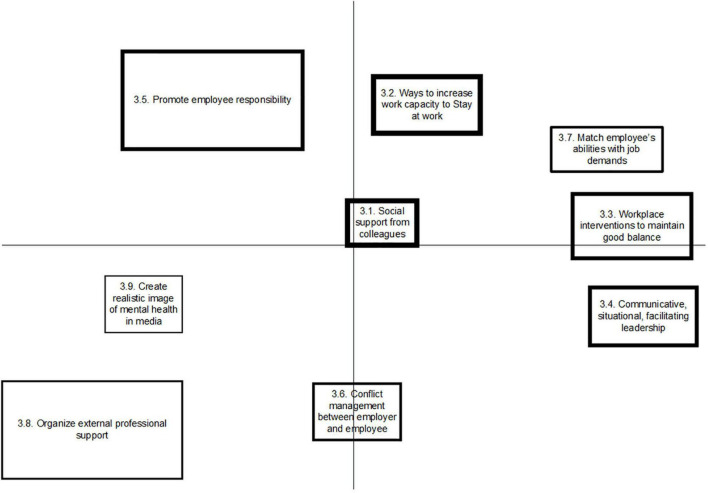
Concept map on factors promoting Stay at work, representing the perspective of occupational health professionals (OHPs).

An overview of the generated clusters per stakeholder group is depicted in Figures 1–3. Each box on the figure visualizes the statements that were placed close to each other, reflecting a cluster. Clusters that are closer together generally have similar concepts. The more distal a cluster is placed on the horizontal or vertical dimension, the more consistency was given to this by the group, referring to a common concept that differs from the other clusters. The mean importance score of each cluster was also calculated. The thicker the line around the cluster, the higher mean importance score was given to it. The five statements that were scored as most important as promoting factors to SAW by each group are presented in this paper, referring to Table 2. A full overview of statements is written out in the Supplementary Material.

**TABLE 2:** The five most important statements and mean importance score ($\bar{n}$) per stakeholder group.

Employees	$\bar{n}$	Supervisors	$\bar{n}$	OHPs	$\bar{n}$
11. Employee is appreciated and seen as valuable by others in the workplace (Cl.1.5)	4.17	43. Teams work in an open and safe climate where there is no judging (Cl.2.7)	4.41	37. Job accommodations and autonomy are used to enable employee to stay in work (Cl.3.2)	4.31
17. Employee feels they have the freedom to set boundaries, for whatever reasons (Cl.1.1)	4.06	6. Supervisor has a people-oriented management style (approachable, accessible, sincere, and transparent) (Cl.2.3)	4.41	10. Receiving support from colleagues (Cl.3.1)	4.23
18. Employee feels they have a healthy workload: has enough to do but no continuous work pressure (Cl.1.2)	3.89	24. Supervisor listens and reflects well with employee about what they observe in the employee’s behavior (Cl.2.3)	4.18	39. Supervisor and employee keep in touch, in the case of reduced work performance or reduced attendance at work (Cl.3.2)	4.15
36. Supervisor offers safety, understanding, a listening ear, avoids judgments and contributes ideas without imposing a solution (Cl.1.4)	3.82	35. Employee is happy in their job and is motivated (Cl.2.2)	4.06	9. Employer creates safe working climate where mental health can be discussed (Cl.3.3)	4.00
48. Supervisor asks what the employee needs, and follows through on agreements about those needs (Cl.1.4)	3.78	16. Supervisor takes symptoms seriously by taking immediate action through conversations and referral (Cl.2.3)	4.00	51. Supervisor makes clear work agreements with employee about expectations, tasks, and job accommodations (Cl.3.4)	3.92

The group of employees with CMHP produced 57 statements that resulted into nine clusters, with arithmetic mean scores of importance ranging from 2.49 to 3.61, referring to Figure 1. The group of supervisors produced 51 statements, which were sorted into 10 clusters, with arithmetic mean scores of importance of clusters ranging from 1.82 to 3.43, referring to Figure 2. Occupational health professionals produced 60 statements, which were sorted into nine clusters, with arithmetic mean scores of clusters ranging from 2.31 to 3.96, referring to Figure 3.

### Meta-Clusters and Clusters

#### Meta-Cluster A: Employee’s Experience of Autonomy in Work

Meta-cluster A contains five clusters reflecting the employee’s experience of autonomy in work, referring to Table 3. Employees created three clusters, the first comprising statements about the employee’s experience of autonomy (Cl.1.1) and the second regarding the employee’s self-regulation and behavior (Cl.1.3). For example, employees who are affected by CMHP in their work value a sense of freedom to exert control over their own tasks, (physical) work environment, or working hours. The third cluster refers to the experience of meaningful work and identity (Cl.1.7). Supervisors identified one, yet highly important cluster reflecting the employee taking responsibility to address issues and find solutions, within a safe working climate (Cl.2.2). OHPs stressed on one similar cluster, regarding the importance of the employee’s sense of responsibility to maintain or increase work participation (Cl.3.5).

**TABLE 3:** Clusters of statements, sorted per meta-cluster, (generated per stakeholder group and given mean importance score per cluster).

Meta-cluster and clusters (EM = employee, SV = supervisor, OPH = occupational health professional)	$\bar{n}$ (mean) importance
**Meta-cluster A: Employee’s experience of autonomy in work**	
1.1 Employee experiences autonomy (EM)	3.61
1.3 Employee self-regulation and behavior (EM)	3.46
2.2 Employee takes responsibility within a safe working climate (SV)	3.37
3.5 Promote sense of responsibility of employee (OHP)	3.06
1.7 Meaningful work and identity (EM)	2.97
**Meta-cluster B: Supervisor support**	
2.1 Supervisor acts proactively, and coaches employee (SV)	3.43
2.3 Supervisor’s leadership style, connected and involved with employee (SV)	3.17
3.4 Communicative, situational, facilitating leadership (OHP)	3.10
1.4 Safe and trusting relationship with supervisor (EM)	3.07
**Meta-cluster C: Ways to match work to individual capacity and needs**	
3.2 Ways to increase capacity to SAW (OHP)	3.54
1.2 Tailored and manageable workload (EM)	3.31
1.6 Realistic work package (EM)	2.98
3.7 Match employee’s abilities with job demands (OHP)	2.94
2.9 Supervisor has practical knowledge of interventions (SV)	2.53
**Meta-cluster D: Safe social climate in workplace**	
3.1 Social support from colleagues (OHP)	3.96
3.3 Workplace interventions to maintain good balance (OHP)	3.26
1.5 Socially safe climate in workplace (EM)	3.03
2.4 Transparent organizational culture (SV)	3.00
2.7 Collective responsibility and appreciation in teams (SV)	2.71
2.8 Employer facilitates a safe work environment (SV)	2.55
3.9. Create realistic image of mental health in media (OHP)	2.31
2.10 Supervisors deal with diversity in team (SV)	1.82
**Meta-cluster E: Professional and organizational support**	
2.5 Support by occupational health social worker (SV)	2.94
1.8 Support outside the workplace (EM)	2.84
2.6 Collaboration between occupational health service and employer (SV)	2.74
3.8 Organize external professional support (OHP)	2.60
1.9 Organizational preconditions (employer) (EM)	2.49
3.6 Conflict management between employer and employee (OHP)	2.27

#### Meta-Cluster B: Supervisor Support

Meta-cluster B “supervisor support” contains four clusters, which all pertain to the supportive role of and relationship with the supervisor, referring to Table 3. Employees formed one cluster, emphasizing on a safe and trustful relationship with the supervisor (cl.1.4), comprising statements such as the supervisor offers a listening ear, avoids judgments, and asks what the employee needs in order to continue working. Two clusters represent the perspective of the supervisors, in which the first cluster concerns the supervisor’s ability to act proactively and coach the employee with CMHP (Cl.2.1). The second cluster is about the supervisors’ leadership style, in which the supervisor shows an involved and connected attitude toward the employee, by taking signals seriously and by listening and reflecting well with the employee about what they observe in the employee’s behavior (Cl.2.3). In line with the supervisors, OHPs identified one cluster regarding the role of the supervisor, regarding the use a communicative, situational, facilitating leadership style (cl.3.4).

#### Meta-Cluster C: Ways to Match Work to the Employee’s Capacity and Needs

Meta-cluster C reflects ways and interventions to match the work or work environment to the employee’s capacities and needs during the time that employees struggle with mental health problems in their work. Employees considered a tailored and manageable workload (cl1.2) as highly important as well as a realistic work package that is genuine and workable (cl.1.6). Supervisors emphasized that having practical knowledge about ways to intervene (cl.2.9) helps to promote SAW among employees with CMHP. Also, according to OHPs, two clusters of statements were created, one comprising ways to increase the employee’s work capacity to SAW (Cl.3.2) and the other cluster is about matching employee’s abilities with job demands (cl.3.7), ideally through consultation of the OHP on the use of job accommodations and autonomy.

#### Meta-Cluster D: Safe Social Climate in the Workplace

Meta-cluster D “safe social climate in workplace” contains eight clusters, referring to Table 3. According to employees with CMHP, one cluster (cl.1.5) reflected the importance of a socially safe climate, containing statements about how employees can be appreciated and valued in their contributions to the work, and about open culture at the workplace. From the perspective of the supervisors, a transparent organizational culture was mentioned to be important (cl.2.4). Besides, two clusters refer to the collective responsibility and appreciation in teams (cl.2.7), and facilities offered by the employer for a safe work environment (cl.2.8). More central on the map (and therefore having less consensus among the group), supervisors named their ability to deal with diversity in teams (cl.2.10). OHPs emphasized on the social support from colleagues (cl.3.1), as well as on the workplace interventions to maintain a good balance for the employee (cl.3.3), such as creating an open culture to discuss mental health and crafting jobs in such a way that there is a variety in tasks. Lastly and with less consensus in the map, OHPs mention the creation of a more realistic image of mental health in the media to reduce stigma and prejudices (Cl.3.9).

#### Meta-Cluster E: Professional and Organizational Support

In meta-cluster E, six clusters were identified, with two clusters from each stakeholder group. Employees produced one cluster representing organizational preconditions from the employer (Cl.1.9) and another cluster referred to the provision of professional support outside of the workplace (Cl.1.8). Supervisors stressed the importance of collaboration between occupational health service and employers (Cl.2.6), e.g., a shared vision on SAW and absenteeism and collaboration to select interventions in a particular case. Another cluster refers to the importance of support from occupational health social workers or peer mentors who are employees with lived experience of CMHP (Cl.2.5). OHPs present clusters, reflecting the organization of external professional support (Cl.3.8), and their own support offered on conflict management between employer and employee (Cl.3.6).

### Differences Between Stakeholders’ Perspectives

The researchers compared the retrieved clusters and statements between the perspectives of employees, supervisors, and OHPs, through thematic analysis. Differences were given mainly in the prioritization task, the responsibility of employee, and formulation by stakeholders. Employees rated statements within meta-cluster A (employee’s role) as important, followed by a safe social climate at the workplace (meta-cluster D) and a trustful relationship with their supervisor (meta-cluster B). Supervisors mostly emphasize the employee’s role (meta-cluster A) as well as their own role (meta-cluster B), showing a joint responsibility of both employer and employee to promote SAW. Supervisors acknowledged the importance in their own role to support employees who face difficulties at work due to CMHP. OHPs rated the psychosocial working climate (meta-cluster D) and ways to match employee’s capacity to the work (meta-cluster C) as highly important. Interestingly, none of the stakeholder groups found clusters about professional and organizational support (meta-cluster E) most important relative to the other clusters; however, it was mentioned by every group through various statements.

Differences were also found through thematic analyses in the interpretations between stakeholders toward the responsibility of the employee. On the one hand, employees value a sense of freedom to exert control over their own tasks, (physical) work environment, or working hours. They stress the importance of being given control and responsibility within their work, especially when employees are struggling at work due to mental health problems. Supervisors, on the other hand, stress the employee’s sense of responsibility to address their issues, especially when mental health problems have an impact on their work. Also, towards the importance of workload, differences were reported. Employees explicitly state a manageable workload and realistic working package on team and organizational level as promoting factors to SAW, while OHPs and supervisors emphasize on adjustments to the individual’s work capacity that may be reduced due to CMHP, through the offer of individual interventions or job accommodations (e.g., the reduction of tasks or responsibilities). Finally, yet importantly, only OHPs mentioned the importance of collegial, practical support, while supervisors and employees named more, in general, a safe working climate, with collective responsibilities and appreciation in teams.

## Discussion

This study represents a conceptualization on how to promote SAW for employees with CMHP from the perspective of a multiple stakeholder. Five meta-clusters were identified based on clusters and statements perceived by medium and highly educated Dutch employees with CMHP, supervisors, and OHPs. The large number and wide variety of statements confirm the dynamic and complex nature of staying at work with CMHP. Despite the different roles that stakeholders have in the workplace, perspectives on promoting factors to SAW overlapped strongly between them. However, differences were found between stakeholder-groups on the rated importance to these shared ideas. Our findings emphasize on a joined responsibility of employee and employer. The employee takes responsibility and autonomy when being treated respectfully for their expertise of their own situation and ideas to SAW. Our study reveals the significant role and responsibility the employer plays, in which workplace stakeholders such as managers have a direct impact on wellbeing of employees and organizational systems that create socially safe workplaces.

First, the presented conceptualization provides novel insights into the relative importance of previously reported, supporting factors from a multiple–stakeholder perspective. These factors act on both individual level, such as personal resources and treatment, reflected in meta-clusters A and E, respectively, and organizational level, such as job demands, job control, co-worker support, supervisor support, job security, reflected in meta-clusters B and C, respectively. This prioritization shows that the main focus should be given to the employee’s experience of work, by addressing one’s qualities, capacities, and needs resulting in the preservation of one’s sense of autonomy and responsibility in work, especially when struggling at work due to CMHP (Bosma et al., 2020). Also, it is crucial to focus on the balance experienced in one’s work, besides the focus on recovery of mental health complaints. Therefore, work should be matched to the employee’s capacity and needs through (timely and temporarily) work or workplace accommodations, as suggested by the literature in other health conditions as well (McDowell and Fossey, 2015; Pak et al., 2019; van der Mei et al., 2021). These work or workplace accommodations could differ as per situation, for example, in case of the COVID-19 pandemic that led to the restriction of work from home. Employees facing mental health issues could be exempted and allowed to work from their workplace office. Each meta-cluster has its own function and cannot act without the others. Meta-clusters A and B reflect the direct interaction between employee and employer, while the other meta-clusters act as conditions to promote SAW. To offer tailored and successful support, it is important to assess the interaction of these meta-clusters, caused by the vast complexity of promoting SAW.

Second, our study revealed that the sense of autonomy in work is highly valued by employees who struggle at work due to CMHP. This is reflected by “being heard or being asked about needs” (autonomy, exerting control) and “being enabled to continue working’ (employer facilitates), theorized by the Capability-for-work model (van der Klink et al., 2016; van Hees et al., 2021b). Interestingly, the literature shows that most interventions aim to strengthen personal and work factors such as coping style, severity of complaints, or job demands, rather than autonomy in work or freedom of choice to SAW (van Hees et al., 2021b). Studies on other (chronic) health conditions also report self-control, autonomy, and freedom of choice as motivators and success factors to SAW (de Vries et al., 2012; Bosma et al., 2020). That autonomy might be more challenging to stimulate among employees with CMHP, may be because lack of control is a common manifestation of CMHP (van der Klink and van Dijk, 2003). Due to stigma (Brouwers et al., 2020) or lack of skills (Janssens et al., 2021), supervisors and OHPs easily tend to take over control, while our study shows the importance of experiencing a sense of autonomy in the SAW process. We recommended OHPs and supervisors to stimulate the autonomy of employees and to address their capacities, by (1) encouraging active participation, (2) asking the right questions, (3) listening to their needs, and (4) supporting SAW as much as possible in order to prevent negative work outcomes. In line with the Self-Determination theory, we underscored that autonomy is one of the psychological needs that facilitates motivation, for any human being and also in the life domain of work (Deci and Ryan, 2008). As jobs of medium and highly educated employees may seem more flexible in terms of workplace accommodations and given autonomy in the job, to stimulate autonomy may seem more applicable for those employees. However, a similar study conducted by our research group among employees with common mental health disorders and low socioeconomic status (SES) showed that potential facilitators to SAW were self-awareness, job control regarding work content and working conditions, and a supportive manager (Vossen et al., 2021). We argue that the organizational systems need to make more effort to facilitate autonomy, in every type of employee or type of job.

Considering the plethora of factors promoting SAW, a recent realist review study showed, based on the Capability-for-work model, how both work-related resources and personal resources, such as cultural background, health status, and coping contribute to positive work outcomes (van Hees et al., 2021b). Work outcomes, such as staying at work and work performance can be realized by the way employees are able or enabled by their workplace to convert these resources into tangible work capabilities such as having meaningful relations at work and exerting control over one’s work (van Hees et al., 2021b). Interestingly, our study showed mostly work-related factors and factor about matching personal life to working life promote SAW. This may suggest that personal factors such as severity of symptoms and previous life experiences may not relate to this work outcome, while previous research has shown they do. In addition, we asked for promoting, enabling factors to SAW and this may leave out hindering factors, formulated as barriers, such as bullying (Nielsen et al., 2016; van Hees et al., 2021b). Although this study is one piece of the puzzle, it stresses the importance of workplace-related factors such as support from the supervisor and a safe social climate. Moreover, these are the factors that are rather tangible and changeable by workplace stakeholders, given their own practical strategies reflected by the statements in this study. Those may inform employers on how to uptake their responsibility and commitment so that their organization can support employees with mental health problems to thrive.

Next, our study shows novel insights on the importance of the interaction between employee and employer, in which the process of SAW takes place. Supervisor support, based on a trustful relationship of communication, is perceived as highly important, besides professional and organizational support that needs to be arranged by the employer (Ketelaar et al., 2017). This interaction should occur in a psychosocial safety climate, to promote SAW. This supportive interaction has been found in previous studies to lead to better mental health and positive work outcomes (Kuoppala et al., 2008; Petrie et al., 2018). Mechanisms and conditions on the interpersonal level deem to be an important addition resulting from this study. From the realist research paradigm, these refer to the relationships between individuals and groups that influence interpretation, reasonings, and use of (workplace) resources in social dynamics (Punton et al., 2016; van Hees et al., 2021a). Therefore, we propose to add interpersonal dynamics as an additional level besides factors on the individual and organizational level (Rugulies and Aust, 2019). Consequently, supervisors and OHPs should address and evaluate the employee’s experience of mechanisms on the interpersonal level, such as perceived support, value and respect, trust, and safety (D’Amato and Zijlstra, 2010; Harvey et al., 2017; Vossen and Van Gestel, 2019; van Hees et al., 2021b).

As clearly stated by all stakeholders in our study, the employer plays a crucial role in enabling employees with CMHP to continue working, endorsing a shared responsibility of all stakeholders (Bosma et al., 2020). On a distal layer at the work floor, efforts by supervisors do not only reflect technical, rather practical skills, such as offering time for individual treatment or reducing job demands or working hours (McDowell and Fossey, 2015). Efforts comprise more “soft” skills by supervisors, in which our participants stated specific strategies to support proactively yet empathically. These statements could contribute to practical guidelines for supervisors on increasing awareness, skills on matching work toward employees’ needs and abilities, offered through training by OHPs. On a proximal layer, more interventions, initiated and implemented by organizations and senior managers, are needed to increase mental health literacy (Moll, 2014; Vossen and Van Gestel, 2019), e.g., through positive psychology in organizations (Van Woerkom et al., 2021). A multilayered strategy may cultivate a culture of support and influence successful implementation (Rycroft-Malone et al., 2004). This is necessary to address the unique context of the work environment that can act as a facilitator to continue working, rather than as a barrier (Moll, 2014).

By giving voice to employees with CMHP, we noticed that a lot can be learned from these employees who succeeded to SAW (Boot and Bosma, 2020). Most participants had gone through the stages of struggling at work, being on sick leave, returning to work, and managing to SAW, possibly making them more efficacious and positive about their ability to work (McGonagle et al., 2014). During the group discussions, participants reported that they felt the importance to improve the employer’s capacity to support employees with CMHP. We underlined this and encouraged participants to interact with colleagues and supervisors about their experiences in their workplaces to fight stigma about mental health in the workplace. Besides, we will use participative processes to develop and evaluate practical guidelines to enhance the capacity of employers in supporting employees with CMHP to continue working (Ketelaar et al., 2017; Van Woerkom et al., 2021).

### Strengths and Limitations

The strength of this study is that each stakeholder group formulated promoting factors from their own perspective, preventing possible conflicting roles interfering the given statements. This turned out to be particularly useful during the focus groups discussions, where participants could freely reflect and deepen the discussion to refine clusters. In addition, the anonymous scoring of the relative importance of perceived factors supports employers to gain insight into what is found to be most important to act upon. By repeating this procedure in various groups and therefore robustly matching the various stakeholders involved in the workplace, we worked toward a more fully saturated picture, increasing external validity (van Bon-Martens et al., 2017).

There are some limitations to this study. First, the study sample consisted of participants with a medium and high educational background, missing out on participants with a low educational background. This may seem comprehensible for supervisors and OHPs, however not for employees with CMHP. An explanation could be that we did not use words that resonate with those employees (e.g., not being fit, not recharged after holiday, continuously feeling low). In response to this limitation, we conducted another study, with additional effort to recruit participants with a lower education background in which we used a different, less linguistic, method to collect data (Vossen et al., 2021). Additional attention is needed in research for minority groups such as migrants or refugees in the work force, who face mental health problems as well as other vulnerabilities, in order to reduce health and workplace inequalities (Gracia et al., 2016; Vossen et al., 2021). Second, it was hard to include employees who were struggling with CMHP, yet staying at work for the first time. This could be due to privacy reasons or due to the unawareness of signals referring to CMHP. Nevertheless, most participating employees with CMHP had extensive experience and were able to translate these experiences to concrete statements; this may be due to the fact that from the various recruitment strategies, most employees were recruited through the route of the activism organizations representing people with CMHP. Third, although we included multiple workplace stakeholders, we did not include colleagues of employees with CMHP, who also play a significant supportive role (Dunstan and Maceachen, 2014).

Based on the abovementioned insights, we suggest to address the following in future research: (1) explore the perspectives of colleagues working with employees with CMHP, (2) explore mechanisms on employee’s work capabilities and (freedom of) choice to SAW, and (3) investigate the relationship between leadership and the prevention of negative work outcomes in employees with CMHP.

## Conclusion

This study offers a conceptualization of SAW, in which multiple workplace stakeholders (Dutch employees with CMHP with medium and high educational backgrounds, supervisors, and OHPs) present similar promoting factors to SAW for employees with CMHP. In addition to organizational and individual efforts, more attention is needed toward the interpersonal dynamics between employer and employee, reflected by tailored work, enhancing autonomy, the employer’s responsibility, and professional support. Above all, a safe and trustful work environment, in which employee’s autonomy, needs, and capacities are addressed by the supervisor, forms a fundamental base to SAW. Our study fills an important gap between theory and practice by presenting strategies and its relative importance for different stakeholders to effectively promote SAW. Results from our study provide practical implications for developing and evaluating such interventions for employers. Because the nature of staying at work is a multifactorial and dynamic process, we suggest that including employees with CMHP and their supervisors is key to planning, evaluation, and implementation of workplace interventions.

## Data Availability Statement

The raw data supporting the conclusions of this article will be made available by the authors, without undue reservation.

## Ethics Statement

The studies involving human participants were reviewed and approved by the Tilburg University (EC-2019.30, date 9 April 2019). The patients/participants provided their written informed consent to participate in this study.

## Author Contributions

SH and BC performed the data collection and analysis. SH was primarily responsible for the draft of the manuscript. All authors reviewed the data analysis, contributed to the writing and revision of the manuscript, study conception and design, and read and approved the final manuscript.

## Conflict of Interest

The authors declare that the research was conducted in the absence of any commercial or financial relationships that could be construed as a potential conflict of interest.

## Publisher’s Note

All claims expressed in this article are solely those of the authors and do not necessarily represent those of their affiliated organizations, or those of the publisher, the editors and the reviewers. Any product that may be evaluated in this article, or claim that may be made by its manufacturer, is not guaranteed or endorsed by the publisher.

## Footnotes

1 Mensen met mogelijkheden, MIND en Samen sterk zonder stigma.

2 MKB Midden Nederland, AWVN.

3 Center for Labor Expertise, Nijkerk, Netherlands.
