# Supplementary material for: Promoting Factors to Stay at Work Among Employees With Common Mental Health Problems: A Multiple-Stakeholder Concept Mapping Study
Source: Front Psychol. 2022 May 10;13:815604. doi: 10.3389/fpsyg.2022.815604 (PMC9128844; doi:10.3389/fpsyg.2022.815604)
Supplement: Supplementary file 1 [file Data_Sheet_1.DOCX]

Supplementary file

Table S1. What employees with CMHP need to SAW from perspective of employees (n=18). Clusters and statements.

| Number | Cluster and statements | Mean score |
| --- | --- | --- |
|  | **1.1 Employee experiences autonomy** | 3.61 |
| 17 | Employee feels they have the freedom to set boundaries, for whatever reasons | 4.06 |
| 19 | Employee experiences freedom and autonomy in their work: can control their tasks, (physical) work environment (e.g. work at home) and leave/holiday | 3.61 |
| 33 | Employee can opt for a quiet work environment, preferably not a flex space or open office | 3.17 |
|  | **1.3 Employee self-regulation and behavior** | 3.46 |
| 27 | Employee feels and knows their own boundaries, can say no, evaluates and redirects when having a bad day | 3.76 |
| 31 | Employee knows what they need to relax outside work (sports, meditation, light therapy lamp, daily structure) | 3.56 |
| 22 | Employee has learned through life experience how to deal with situations and to be kind to themselves | 3.47 |
| 25 | Employee proactively takes responsibility by seeking and accepting support (psychologist, family doctor, friends/family) | 3.44 |
| 21 | Employee has insight into their own strengths and weaknesses (managing calendar, stress factors, overestimating them self) | 3.33 |
| 30 | Employee knows and indicates what they need at work, also when struggling (e.g. frequent breaks, noise cancelling headset, a walk during lunch) | 3.22 |
| 13 | Employee is more open about CMHP when they know their supervisor supports them | 2.67 |
| 26 | Employee’s openness depends on the context: trust, work relationship and how receptive the supervisor is | 2.61 |
|  | **1.2 Tailored and manageable workload** | 3.31 |
| 18 | Employee feels they have a healthy workload: has enough to do but no continuous work pressure | 3.89 |
| 42 | Supervisor takes signals seriously by offering preventative measures and discussing solutions to reduce workload | 3.72 |
| 50 | Employee feels that employer takes measures to reduce workload when needed | 3.44 |
| 3 | Employee’s direct colleagues know about employee’s situation and contribute their thoughts, protect the employee when needed | 2.89 |
| 16 | Employee checks that their own perception of the workload aligns with expectations of supervisor and colleagues | 2.59 |
|  | **1.4 Safe and trusting relationship with supervisor** | 3.07 |
| 36 | Supervisor offers safety, understanding, a listening ear, avoids judgements and contributes ideas without imposing a solution | 3.82 |
| 48 | Supervisor asks what the employee needs, and follows through on agreements about those needs | 3.78 |
| 40 | Supervisor knows and encourages employee’s qualities, motivation and ambitions, also when employee is struggling at work | 3.39 |
| 38 | Supervisor gives employee honest feedback about work performance | 3.33 |
| 44 | Supervisor supports the priorities in the employee’s work package, and accommodates a (temporary) workload reduction | 3.28 |
| 39 | Supervisor regularly initiates conversions with employee, asking how things are going | 3.06 |
| 43 | Supervisor picks up on problems during personal conversation, without judging | 3.00 |
| 46 | Supervisor genuinely shows personal interest in employees, without immediately starting to talk about work matters | 2.88 |
| 47 | Supervisor trusts employees in the work they do | 2.83 |
| 45 | Supervisor activates employee to take action when they notice changes in behavior (perfectionism, frequent absence or overcompensation) | 2.78 |
| 41 | Supervisor also shows their own vulnerabilities | 2.50 |
| 49 | Supervisor asks in annual performance appraisal about potential non-work issues that might affect work | 2.24 |
|  | **1.5 Safe social climate in workplace** | 3.03 |
| 11 | Employee is appreciated and seen as valuable by others in the workplace | 4.17 |
| 8 | Workplace offers a safe atmosphere and open culture (transparent communications, no gossip, feeling safe) | 3.61 |
| 9 | Others in the workplace give employee compliments and positive encouragement, not patronizing | 3.56 |
| 35 | Employee feels indispensable and feels acknowledged when at work, even when they are struggling | 3.44 |
| 2 | There is trust between colleagues, and the employee can say what they want to say, under all circumstances | 3.39 |
| 1 | Colleagues are open about difficult tasks/situations in their work and support each other in these | 3.28 |
| 10 | Employer gives positive, clear communication where colleagues stick to work agreements | 3.22 |
| 7 | Employee has a click with at least one colleague, who more regularly asks how they are doing | 3.17 |
| 4 | Employee has fun at work and works within a close-knit team where there is space for non-work matters | 2.78 |
| 52 | Employee feels differences between team members are accepted at all layers of the organization, also vulnerabilities | 2.78 |
| 6 | Colleagues update each other regularly on how things are going and support each other | 2.61 |
| 28 | Employee knows they are not the only one with mental health issues | 2.33 |
| 29 | Employee shares experiences with others at work; this gives a sense of recognition | 1.94 |
| 53 | Employer offers a safe platform for employees who experience CMHP and there is attention for vulnerability in the workplace | 2.61 |
|  | **1.6 Realistic work package** | 2.98 |
| 37 | Supervisor and employee openly discuss whether the work package, work environment or work level is realistic and suitable for the long term | 3.11 |
| 34 | Employee is (temporarily) relieved from certain tasks/administration and can focus on clearly defined tasks | 3.06 |
| 15 | Employee (temporarily) performs work that gives instead of drains energy, but work content remains at the same intellectual level | 2.76 |
|  | **1.7 Meaningful work and identity** | 2.97 |
| 20 | Employee feels their work is important and meaningful | 3.47 |
| 32 | Work provides the employee with distraction from CMHP and contributes to identity and self-confidence | 3.28 |
| 5 | The team has a shared goal/vision to which the employee contributes together with their colleagues | 2.17 |
|  | **1.8 Professional support outside the workplace** | 2.84 |
| 56 | Employee receives tailored professional support, with a stable treatment relationship | 3.61 |
| 24 | Employee can reflect on problematic issues with a confidential counsellor/ independent coach | 2.53 |
| 55 | Professional support also addresses work-related issues and the employee can take steps to work on these | 2.39 |
|  | **1.9 Organizational preconditions (employer)** | 2.49 |
| 57 | Occupational health physician, occupational health and safety service and HR take complaints seriously and communicate on a basis of trust and empathy | 3.35 |
| 51 | Employer has vision and policy on healthy work practices / working conditions | 2.67 |
| 12 | Employee is given clarity about contract (permanent or clarity about whether or not contract will be extended) | 2.47 |
| 54 | Employer intervenes if there are long waiting times for treatment | 2.00 |
| 23 | Employee can take courses and training such as mindfulness, stress management | 1.94 |

Table S2. What employees with CMHP need to SAW from the perspective of supervisors (n=17). Clusters with statements.

| Number | Cluster and statements | Mean score |
| --- | --- | --- |
|  | **2.1 Supervisor acts proactively and coaches employee** | 3.43 |
| 28 | Supervisor asks what employee is still able to do despite CMHP-related issues | 3.94 |
| 21 | Supervisor encourages employee to stay at work for distraction, daily structure and self-esteem | 3.82 |
| 27 | Supervisor asks what employee needs to continue working | 3.71 |
| 22 | Supervisor encourages employee to take steps in their private life, alongside job accommodations | 3.53 |
| 29 | Supervisor points out the employee’s own responsibility, encourages employee to find solutions themselves | 3.18 |
| 15 | Supervisor and employee make clear work agreements and communicate those to team members | 2.94 |
| 20 | Supervisor quickly suggests (temporary) job accommodations to give employee space to work on problems | 2.76 |
| 26 | Supervisor acts as a buffer to protect employee from problems in the organization (staff shortage, reorganization, procedures) | 2.12 |
|  | **2.2 Employee takes responsibility within a safe working climate** | 3.37 |
| 35 | Employee is happy in their job and is motivated | 4.06 |
| 37 | Employee takes responsibility by accepting support and seeking solutions | 4.00 |
| 32 | Employee dares to open up and show their vulnerability, or is coached to do this | 3.88 |
| 19 | Supervisor identifies CMHP-related changes in the employee’s behavior early on | 3.82 |
| 34 | Employee is highly committed to the organization or their work, but does not feel indispensable in the case of illness or CMHP | 3.24 |
| 33 | Employee has or aims to have a healthy lifestyle | 2.94 |
|  | **2.3 Supervisor’s leadership style: connected and involved with employee** | 3.17 |
| 6 | Supervisor has a people-oriented management style (approachable, accessible, sincere and transparent) | 4.41 |
| 24 | Supervisor listens and reflects well with employee about what they observe in the employee’s behavior | 4.18 |
| 16 | Supervisor takes symptoms seriously by taking immediate action through conversations and referral | 4.00 |
| 23 | Supervisor shows appreciation for all employees, including those who are less visible in their work | 3.65 |
| 10 | Supervisor has insight into the employee’s work motives | 3.59 |
| 18 | Supervisor reacts to signals of short-term sick leave | 3.53 |
| 11 | Supervisor is transparent and honest about career perspectives, which can prevent mental health problems | 3.29 |
| 4 | Supervisor evaluates conversations with employee and reflects on themselves to improve their conversation techniques | 3.18 |
| 25 | Supervisor converses attentively with employees outside the direct workplace | 3.12 |
| 9 | Supervisor increases frequency of contact when employees struggle at work | 2.94 |
| 3 | Supervisor pays extra attention to employees with high risk of CMHP (overambitious/insecure/introverted) | 2.88 |
| 13 | Supervisor knows what the employee’s job involves and so can assess the employee’s performance properly | 2.71 |
| 14 | Supervisor shows their own (mental) vulnerability or weaknesses | 2.71 |
| 8 | Supervisor has some knowledge about mental health | 2.59 |
| 7 | Supervisor has a good relationship with the employee, built up over the time they have worked together | 2.41 |
| 1 | Supervisor clearly limits their role to work-related issues and refers to professionals for private issues | 2.00 |
| 31 | Supervisor makes extra effort if the employee has built up a lot of credit in the past | 1.59 |
|  | **2.4 Transparent organizational culture** |  |
| 51 | Organization is open about mental health | 3.00 |
| 47 | Supervisor or employee informs direct colleagues about issues, also CMHP-related issues | 2.82 |
|  | **2.8 Support by occupational health social worker or peer mentor** |  |
| 17 | Supervisor organizes support from company social worker or co-workers with lived experience of CMHP supporting as peer mentor | 2.94 |
|  | **2.6 Collaboration between occupational health service and employer** |  |
| 50 | Occupational health physician and supervisor collaborate to identify suitable job accommodations so employee can stay at work | 3.71 |
| 49 | Occupational health service and employer share vision on sustainable work participation | 2.94 |
| 40 | Employer offers training and education about working with CMHP | 2.59 |
| 52 | Employees who are having a hard time at work are discussed in multidisciplinary meetings between the organization and occupational health service | 2.47 |
| 48 | Employer appoints a case manager to maintain contact with external parties | 2.00 |
|  | **2.7 Collective responsibility and appreciation in team** | 2.71 |
| 2 | Supervisor appreciates team performance, also when colleagues support one another | 2.71 |
| 43 | Teams work in an open and safe climate where there is no judging | 4.41 |
| 42 | Team and supervisor develop a collective vision on team climate, illness and sick leave | 3.24 |
| 44 | Team has a sense of joined responsibility that makes vulnerable employees feel supported | 3.24 |
| 45 | Team: colleagues identify behavioral changes related to CMHP and communicate this to the colleague and supervisor concerned | 3.06 |
| 46 | Team: clarity about work agreements and schedules, tailored to the needs of vulnerable employees | 2.82 |
|  | **2.8 Employer facilitates a safe work environment** | 2.55 |
| 41 | Organization has an independent coach/confidential adviser who employees can talk to in a safe and open environment | 3.18 |
| 38 | Organization offers a safe space (coffee corner, break room) where colleagues can meet and talk | 2.29 |
| 39 | Organization offers quiet or low-stimulus work spaces for vulnerable employees | 2.18 |
|  | **2.9** **Supervisor has practical knowledge of interventions** |  |
| 12 | Supervisor is aware of and actively offers interventions or job accommodations that promote mental health | 3.24 |
| 30 | Supervisor encourages mobility or (temporarily) placement of employee with another department or team if CMHP is work related | 1.82 |
|  | **2.10 Supervisor deal with diversity in team** | 1.82 |
| 53 | Supervisor encourages lower-educated employees to find solutions to their problems (in work and home life) | 1.82 |

Table S3. What employees with CMHP need to SAW from the perspective of occupational health professionals (n=13). Clusters and statements.

| Number | Cluster and statements | Mean score |
| --- | --- | --- |
|  | **3.1 Social support from colleagues** | 3.96 |
| 10 | Receiving support from colleagues | 4.23 |
| 11 | Good and warm atmosphere among colleagues | 3.69 |
|  | **3.2 Ways to increase work capacity to SAW** | 3.54 |
| 37 | Job accommodations and autonomy are used to enable employee to stay in work | 4.31 |
| 39 | Supervisor and employee keep in touch, in the case of reduced work performance or reduced attendance at work | 4.15 |
| 14 | Employee perceives their work as meaningful and work energizes them | 3.54 |
| 13 | Employees feel indispensable and valued in their work | 2.15 |
|  | **3.3 Workplace interventions to maintain good balance** | 3.26 |
| 9 | Employer creates safe working climate where mental health can be discussed | 4.00 |
| 32 | Supervisor offers job accommodations, e.g. in goals, targets, pace, tasks, stimuli and physical work environment | 3.54 |
| 33 | Employer offers varied tasks so employee can vary switch between tasks when they get stuck on certain tasks. | 3.38 |
| 25 | Supervisor encourages employee to continue working if the source of CMHP is not work (e.g. stress from home) | 3.31 |
| 12 | Adjust physical work environment based on the needs of employee (e.g. noise, amount of stimuli in open office) | 2.85 |
| 18 | Supervisor gives feedback on employee’s efforts | 2.46 |
|  | **3.4 Communicative, situational, facilitating leadership** | 3.10 |
| 51 | Supervisor makes clear work agreements with employee about expectations, tasks and job accommodations | 3.92 |
| 53 | Supervisor recognizes signals or behavioral changes, related to CMHP | 3.62 |
| 56 | Supervisor intervenes on time when they can identify signs of mental health issues (conversation, intervention) | 3.62 |
| 3 | Higher management prioritizes sustainable employability and facilitates supervisors to practice health-promoting leadership | 3.54 |
| 49 | Employer invests in supervisors, especially in terms of them being able to identify CMHP | 3.54 |
| 58 | Supervisor is sufficiently competent to discuss mental health (issues) with employee (e.g. coaches, offers safety and asks questions). | 3.54 |
| 54 | Supervisor is honest about job performance, even if there are doubts or signals of reduced performance | 3.46 |
| 55 | Supervisor is aware that CMHP can vary, and adjusts workload accordingly | 2.92 |
| 59 | Supervisor strikes a balance between encouraging employee autonomy and addressing responsibilities | 2.85 |
| 52 | The supervisor knows the employee and talks to them about the meaning/value of work | 2.69 |
| 15 | Employee allows colleagues or supervisor to alert them to changes in their behavior | 2.69 |
| 57 | Supervisor is familiar with interventions for mental health and recovery | 2.62 |
| 30 | Employer provides support outside the workplace, in the form of care leave and financial assistance | 2.23 |
| 60 | When changes occur at the workplace, the supervisor informs employees at an early stage, especially vulnerable employees | 2.23 |
|  | **3.5 Promote employee responsibility** | 3.06 |
| 24 | Employee has knowledge and insight into their own (CMHP-related) issues and behavior | 3.85 |
| 23 | Employee has a healthy attitude towards their work, and has a good work-life balance | 3.54 |
| 8 | Employee can communicate with colleagues about how best to deal with them and their limitations | 3.46 |
| 16 | Employee is prepared to accept and receive (professional) support/treatment if mental health complaints lead to limitations | 3.38 |
| 17 | Employee is open about how CMHP affects their work, to a certain extent | 3.38 |
| 38 | Employee stays (partly) at work to maintain daily routine with sufficient distraction and structure, | 3.08 |
| 26 | Employee has a stable home environment | 3.00 |
| 20 | Employee experiences or gets a chance to explore and improve self-management | 2.85 |
| 28 | Social environment in private life shows understanding and gives space | 2.69 |
| 4 | Employee has a healthy life style, or is encouraged to develop this | 2.54 |
| 27 | Employee’s values and norms developed as a child motivate them to stay at work | 1.85 |
|  | **3.7 Match employee’s abilities with job demands** | 2.94 |
| 40 | OHP knows when an employee should stop working (e.g. if work itself is a cause of mental health issues, or if tasks can no longer be modified) | 3.69 |
| 29 | Employee’s job title and tasks should match their educational level (and not be too high) | 3.46 |
| 1 | Tasks and responsibilities should match employee’s stage of life | 3.08 |
| 21 | Employee should receive support if tasks temporarily become too difficult due to CMHP | 2.85 |
| 35 | Supervisor and employee should discuss Mobility, demotion, contract reduction or job accommodations at an early stage to avoid long-term sick leave | 2.69 |
| 31 | Employee’s workload should match their work capacity | 2.62 |
| 34 | Supervisor should not increase employee’s job demands or responsibilities until the employee can manage their current tasks | 2.62 |
| 36 | Supervisor should provide regular recovery periods for vulnerable employees, structurally scheduled or forced despite 24-hour economy | 2.54 |
|  | **3.8 Organize external professional support** | 2.60 |
| 42 | The regular mental health professional discusses work-related topics during employee’s treatment | 3.92 |
| 46 | OHP works closely with employer and knows the work environment | 3.31 |
| 41 | OHP acts together with the supervisor and employee in acute stage to find a practical solution to stay at work | 3.23 |
| 47 | Employer offers supervisor regular low-threshold consultation with OHP about at-risk employee | 3.23 |
| 22 | Employer facilitates low-threshold access to job coach or treatment to help to help the employee learn to cope with CMHP in relation to work | 3.08 |
| 43 | In the case of long waiting times in regular treatment, the employer organizes treatment through OHP | 2.85 |
| 45 | OHP supports the less assertive employee, advising the employer on what the employee needs | 2.31 |
| 19 | Employee is offered support from peer mentor | 2.15 |
| 50 | Employee and supervisor have access to job coach to learn tools that help deal with employee’s problems | 2.08 |
| 6 | Employer has access to knowledge about workplace interventions, available from OHP associations | 1.92 |
| 2 | SMEs have good access to OHP services | 1.62 |
| 5 | Municipalities support employers to keep people in work and prevent sick leave | 1.54 |
|  | **3.9 Create realistic image of mental health in media** | 2.31 |
| 7 | Attention for mental health and work in regular and social media | 2.31 |
|  | **3.6 Conflict management between employer and employee** | 2.27 |
| 44 | In the case of conflict between the employer and employee engage (independent) mediator as soon as possible | 2.77 |
| 48 | In the case of short-term sick leave involve OHP as soon as possible | 1.77 |
